# Risk Factors Associated With Atrioventricular Block

**DOI:** 10.1001/jamanetworkopen.2019.4176

**Published:** 2019-05-24

**Authors:** Tuomas Kerola, Antti Eranti, Aapo L. Aro, M. Anette Haukilahti, Arttu Holkeri, M. Juhani Junttila, Tuomas V. Kenttä, Harri Rissanen, Eric Vittinghoff, Paul Knekt, Markku Heliövaara, Heikki V. Huikuri, Gregory M. Marcus

**Affiliations:** 1 Department of Internal Medicine, Päijät-Häme Central Hospital, Lahti, Finland; 2 Electrophysiology Section, Division of Cardiology, University of California, San Francisco; 3 Division of Cardiology, Heart and Lung Center, Helsinki University Hospital, Helsinki, Finland; 4 Research Unit of Internal Medicine, Medical Research Center, Oulu University Hospital and University of Oulu, Oulu, Finland; 5 The National Institute for Health and Welfare, Helsinki, Finland; 6 Department of Epidemiology and Biostatistics, University of California, San Francisco

## Abstract

**Importance:**

Pacemaker implantations as a treatment for atrioventricular (AV) block are increasing worldwide. Prevention strategies for AV block are lacking because modifiable risk factors have not yet been identified.

**Objective:**

To identify risk factors for AV block in community-dwelling individuals.

**Design, Setting, and Participants:**

In this population-based cohort study, data from the Mini-Finland Health Survey, conducted from January 1, 1978, to December 31, 1980, were used to examine demographics, comorbidities, habits, and laboratory and electrocardiographic (ECG) measurements as potential risk factors for incident AV block. Data were ascertained during follow-up from January 1, 1987, through December 31, 2011, using a nationwide registry. A total of 6146 community-dwelling individuals were included in the analysis performed from January 15 through April 3, 2018.

**Main Outcomes and Measures:**

Incidence of AV block (hospitalization for second- or third-degree AV block).

**Results:**

Among the 6146 participants (3449 [56.1%] women; mean [SD] age, 49.2 [12.9] years), 529 (8.6%) had ECG evidence of conduction disease and 58 (0.9%) experienced a hospitalization with AV block. Older age (hazard ratio [HR] per 5-year increment, 1.34; 95% CI, 1.16-1.54; *P* < .001), male sex (HR, 2.04; 95% CI, 1.19-3.45; *P* = .01), a history of myocardial infarction (HR, 3.54; 95% CI, 1.33-9.42; *P* = .01), and a history of congestive heart failure (HR, 3.33; 95% CI, 1.10-10.09; *P* = .03) were each independently associated with AV block. Two modifiable risk factors were also independently associated with AV block. Every 10–mm Hg increase in systolic blood pressure was associated with a 22% higher risk (HR, 1.22; 95% CI, 1.10-1.34; *P* = .005), and every 20-mg/dL increase in fasting glucose level was associated with a 22% higher risk (HR, 1.22; 95% CI, 1.08-1.35; *P* = .001). Both risk factors remained statistically significant (HR for systolic blood pressure, 1.26 [95% CI, 1.06-1.49; *P* = .007]; HR for glucose level, 1.22 [95% CI, 1.04-1.43; *P* = .01]) after adjustment for major adverse coronary events during the follow-up period. In population-attributable risk assessment, an estimated 47% (95% CI, 8%-67%) of AV blocks may have been avoided if all participants exhibited ideal blood pressure and 11% (95% CI, 2%-21%) may have been avoided if all had a normal fasting glucose level.

**Conclusions and Relevance:**

In this analysis of data from a population-based cohort study, suboptimal blood pressure and fasting glucose level were associated with AV block. These results suggest that a large proportion of AV blocks are assocated with these risk factors, even after adjusting for other major adverse coronary events.

## Key Points

**Question:**

Are there readily modifiable risk factors associated with the risk of atrioventricular block?

**Findings:**

In this population-based cohort study of 6146 community-dwelling individuals, elevated blood pressure and blood glucose levels were associated with the development of atrioventricular block. Population-attributable risk calculations suggest that elevated blood pressure and glucose levels may be associated with more than half of all cases of atrioventricular block.

**Meaning:**

Optimizing blood pressure and glucose level control may serve as effective strategies to prevent clinically relevant conduction disease and pacemaker implantation.

## Teaser

This population-based cohort study identifies modifiable risk factors associated with atrioventricular block among community-dwelling respondents to the Mini-Finland Health Survey.

## Introduction

Atrioventricular (AV) block is a common reason for pacemaker implantation, and the number of pacemaker implantations is increasing.^1^ Atrioventricular block most commonly occurs in the absence of significant cardiac disease and is generally attributed to idiopathic fibrosis of the conduction system.^2^ By definition, the cause of that fibrosis remains unknown.

Less severe conduction abnormalities, such as PR prolongation^3^ and right^4^ and left^5^ bundle branch block (BBB), are known to be associated with more severe forms of AV block requiring a pacemaker. Although hypertension and higher fasting glucose level each predispose to these less severe conduction abnormalities,^6,7,8^ no evidence to date, to our knowledge, suggests that such modifiable risk factors might be connected to AV block itself.

Although pacemakers can usually provide adequate treatment of the symptoms of AV block, no preventive or curative strategies are currently used in clinical practice. Although generally a low-risk procedure, pacemaker implantation can involve a risk of serious complications, such as pneumothorax, cardiac tamponade, and death.^9^ After implantation, patients require generator changes, which carry a particularly high risk of infection and resultant endocarditis.^10^ Finally, independent of these procedural complications, successful pacemaker therapy has been associated with a worse prognosis.^11,12,13^

A better understanding of the conditions associated with severe AV conduction disease would enable the development of prevention strategies, ideally avoiding the pacemaker-associated complications and increased use of health care resources. No previous study, to our knowledge, has reported the population-based characteristics associated with incident AV block. We therefore sought to identify risk factors for AV block, with a particular emphasis on characteristics that are known to be modifiable.

## Methods

### Study Design

Participants were enrolled in the Mini-Finland Health Survey from January 1, 1978, to December 31, 1980, as part of the Finnish Mobile Clinic Survey of the Social Insurance Institution, Helsinki, focusing on the health status of the Finnish population. The detailed study protocol and methods have been previously published.^14,15^ Briefly, the original random sample was representative of the Finnish population in 1978. The study included a health interview performed by a home-visiting trained nurse followed by a health examination at the study clinic. A total of 8000 individuals (3637 men and 4363 women) older than 30 years received an invitation to the survey, of whom 7217 participated in health examinations. Participants were excluded from the current study if they were deceased before ascertainment of AV block occurred, if they had missing or poor electrocardiographic (ECG) data, if they had second- or third-degree AV block or atrial or ventricular pacing on their baseline ECG, or if data pertinent to any of the relevant covariates were missing, leaving 6146 participants for the present study. The Mini-Finland Health Survey preceded the current legislation on ethics in medical research in Finland.^15^ All participants were fully informed about the study and participated in the study voluntarily, and the use of the information for medical research was explained to them. Agreeing to participate in the baseline health examination was taken to indicate written informed consent. The participants were free to unconditionally withdraw their consent at any time, in which case their data were deleted. Record linkage of national health registers to the survey data was approved by the register authorities (the Social Insurance Institution; the National Institute for Health and Welfare, Helsinki; and Statistics Finland, Helsinki). Certification to analyze these deidentified data was provided by the institutional review board of the University of California, San Francisco. This study followed the Strengthening the Reporting of Observational Studies in Epidemiology (STROBE) reporting guideline for cohort studies.

### Demographics and Anthropometric Measurements

Age and sex were self-reported by study participants. Height was directly measured by study personnel. Weight was measured using a calibrated scale with participants in light clothing, and 1 kg was subtracted from the measured result. Blood pressure was measured using a manual sphygmomanometer after 40 minutes of completing questionnaires in a seated and comfortable position. No eating, drinking, or smoking during that 40-minute period was allowed. Two blood pressure measurements were obtained during a 1.5-minute interval, and the latter systolic and diastolic readings were used for the purposes of the present study.

### ECG Measurements

A resting paper ECG was recorded using the Olli 308 ECG device (KONE Oyj), with a paper speed of 50 mm/s and a calibration of 10 mm/mV. All ECGs were classified by 4 trained experts working in pairs using revised Minnesota coding as described previously.^16,17^ Conduction disorders such as incomplete or complete right BBB, left anterior hemiblock, left posterior hemiblock, left BBB, and interventricular conduction delay were manually confirmed for the purposes of the present study according to revised criteria.^18^

### Laboratory Measurements

Blood samples were obtained after 11 hours of fasting. Levels of serum cholesterol, high-density lipoprotein cholesterol, triglycerides, and plasma glucose were measured in a single laboratory using standard laboratory techniques.

### Cardiovascular Comorbidities

Hypertension was defined as systolic blood pressure of greater than 140 mm Hg, diastolic blood pressure of greater than 90 mm Hg, or a self-reported history of hypertension with the use of blood pressure–lowering medication. Diabetes was defined as a fasting glucose level of at least 200 mg/dL in a single measurement or at least 120 mg/dL in 2 separate measurements (to convert to millimoles per liter, multiply by 0.0555) or use of a medication to lower glucose levels. All participants underwent a standard 12-lead ECG and posterior-anterior and lateral chest radiography. Study nurses specifically screened for each of the following diagnoses, which required confirmation by a study physician. Angina pectoris was defined as a history of typical exercise-related chest pain relieved within minutes of rest or with use of nitroglycerin substrates. Myocardial infarction (MI) was defined as present given at least 1 of the following criteria: pathologic Q waves indicating transmural infarction on study ECG; ECG findings consistent with a possible MI and previous hospitalization because of MI with elevated cardiac enzyme levels; or typical history of MI and previous hospitalization because of MI with elevated cardiac enzyme levels. Congestive heart failure (CHF) was defined as present given at least 1 of the following criteria: documented history of treatment of CHF symptoms, such as with diuretics, with a resultant positive response; signs of cardiac decompensation on clinical examination; or significant enlargement of the heart on chest radiographs (>650 cm^3^/m^2^ for men and >600 cm^3^/m^2^ for women).

### Habits

Alcohol consumption and cigarette smoking were assessed using standard questionnaires. Current or past smoking was defined as smoking of cigarettes regularly for a minimum of 1 year. Participants were classified as current smokers, former smokers, or nonsmokers. Self-reported mean intake of beer, wine, and liquor was used to calculate alcohol consumed on a regular basis as grams per week.

### Follow-up Data

Follow-up for hospitalization with second- or third-degree AV block, the primary end point for the current study, began on January 1, 1987, when the use of the *International Classification of Diseases, Ninth Revision (ICD-9)*, was introduced in Finland. The first hospitalization with *ICD-9* codes I44.1 (second-degree AV block) or I44.2 (third-degree AV block) was used as the primary outcome. Major adverse coronary events (MACEs) were defined as death or hospitalization due to acute coronary syndrome (including unstable angina or MI) or a coronary revascularization procedure (*International Classification of Diseases, Eighth Revision,* and *ICD-9* codes 410 or 411; *International Statistical Classification of Diseases and Related Health Problems, Tenth Revision* codes I20.0, I21, or I22; or angioplasty or coronary artery bypass graft). Follow-up for MACEs was performed from the baseline examination until December 31, 2011. Nationwide health registries were the source of all mortality (Statistics Finland) and hospitalization (RegisterCare, Register for Health Care, and National Institute for Health and Welfare) data. This method has been previously validated and shown to capture 94% of all events with no secular variation in accuracy.^19^

### Statistical Analysis

Data were analyzed from January 15 through April 3, 2018. Continuous variables with a normal distribution are presented as mean (SD) and were compared using unpaired 2-tailed *t* tests. Nonnormally distributed continuous variables are presented as medians with interquartile range and were compared using the Mann-Whitney test. Categorical variables are presented as absolute numbers and percentages and were compared using the χ^2^ test.

Cumulative incidence curves taking death as a competing risk into account were constructed to illustrate the associations between covariates and incident AV block. Cox proportional hazards regression models were then used to calculate hazard ratios (HRs) and their 95% CIs for the incidence of AV block. Inclusion of covariates in the multivariable model was first determined by selecting those that exhibited 2-sided *P* < .10 in unadjusted analyses. With sex and age included as fixed covariates in the multivariable model, inclusion of additional covariates was determined by performing a stepwise backward selection process until all the other variables in the model exhibited *P* < .10. To test the reproducibility of the results, we performed a sensitivity analysis wherein we adjusted for baseline demographics and all covariates with *P* < .10 to determine whether the same covariates remained statistically significant.

Because evidence of conduction disease on baseline ECG would more likely mediate rather than confound relationships, we used a separate model including pertinent ECG data. We performed 2 secondary analyses. First, because intervening cardiovascular events may have mediated observed associations, we performed an analysis adjusting for time-updated MACEs. Second, because the pathophysiology of third-degree AV block may be different from that of second-degree AV block, we performed an analysis restricted to third-degree AV block as the outcome.

The population-attributable risk of incident AV block was calculated for modifiable risk factors found to have a statistically significant association with incident AV block using a semiparametric approach.^20,21^ Specifically, the ratio of the mean excess risk associated with the exposure of interest to the mean observed risk was calculated. Reference levels for systolic blood pressure (120 mm Hg) and fasting glucose level (100 mg/dL) were chosen according to recent guidelines,^22,23^ and 95% CIs for these population-attributable risk estimates were obtained using bootstrap resampling with 500 repetitions. To assess these population-attributable risks independent of the association with acute coronary events, we performed a second analysis that omitted participants who had experienced an MACE.

All models fulfilled proportional hazards assumptions. Data were analyzed using SPSS, version 23 (IBM Corp); Stata, version 15 (StataCorp); and R, version 3.5.2 (R Project for Statistical Computing). A 2-tailed *P* < .05 was considered statistically significant.

## Results

The mean (SD) age of the 6146 participants was 49.2 (12.9) years; 2697 (43.9%) were men and 3449 (56.1%) were women. Electrocardiographic evidence of conduction disease was observed in 529 (8.6%) participants. The baseline characteristics according to the presence or absence of infranodal conduction disease are presented in Table 1. Characteristics of participants excluded from the study (n = 1071) because of early death (before January 1, 1987) or missing data are listed in the eTable in the Supplement.

**Table 1.** Baseline Characteristics of Participants With Normal Conduction vs Conduction Abnormality at Baseline

Characteristics	Study Group^a^		*P* Value^c^
	Normal Conduction (n = 5617)	Any Conduction Disease (n = 529)^b^	
Age, y	48.6 (12.7)	55.4 (13.7)	<.001
Male sex, No. (%)	2394 (42.6)	303 (57.3)	<.001
Height, cm	165.7 (9.3)	167.0 (10.1)	.002
Weight, kg	70.9 (12.9)	74.7 (13.5)	<.001
Body mass index^d^	25.8 (4.0)	26.8 (4.3)	<.001
Heart rate, bpm	67.6 (13.2)	66.0 (13.3)	.007
Blood pressure, mm Hg			
Systolic	140.8 (21.6)	147.1 (23.0)	<.001
Diastolic	86.5 (11.3)	87.5 (11.6)	.06
Hypertension, No. (%)	3148 (56.0)	362 (68.4)	<.001
Diabetes, No. (%)	184 (3.3)	41 (7.8)	<.001
Angina pectoris, No. (%)	176 (3.1)	68 (12.9)	<.001
Myocardial infarction, No. (%)	114 (2.0)	46 (8.7)	<.001
Congestive heart failure, No. (%)	85 (1.5)	38 (7.2)	<.001
Blood pressure–lowering medication, No. (%)	664 (11.8)	127 (24.0)	<.001
Alcohol consumption, median (IQR), g/wk	7.0 (0-49)	4.0 (0-36)	.02
Smoking status, No. (%)			
Nonsmoker	3180 (56.6)	293 (55.4)	.77
Ex-smoker	1149 (20.5)	115 (21.7)	
Current smoker	1288 (22.9)	121 (22.9)	
Cholesterol level, mg/dL	267 (54)	271 (54)	.25
High-density lipoprotein cholesterol level, mg/dL	66 (15)	62 (15)	<.001
Triglyceride level, mg/dL	58 (35)	66 (77)	<.001
Fasting glucose level, mg/dL	95 (22)	99 (25)	.001
PR interval, ms	157.9 (21.1)	196.1 (33.1)	<.001
QRS duration, ms	88.6 (10.6)	100.4 (18.8)	<.001

Abbreviation: IQR, interquartile range.

SI conversion factors: To convert cholesterol to millimoles per liter, multiply by 0.0259; glucose to millimoles per liter, multiply by 0.0555; triglycerides to millimoles per liter, multiply by 0.0113.

^a^ Unless otherwise indicated, data are expressed as mean (SD).

^b^ Includes first-degree atrioventricular block (n = 331), interventricular conduction delay (n = 53), left anterior hemiblock (n = 45), left posterior hemiblock (n = 50), incomplete (n = 16) or complete (n = 42) right bundle branch block, and incomplete (n = 2) or complete (n = 16) left bundle branch block.

^c^ Calculated using unpaired 2-tailed *t* test, χ^2^ test, and Mann-Whitney test.

^d^ Calculated as weight in kilograms divided by height in meters squared.

### Characteristics Associated With Atrioventricular Block

During follow-up, 58 individuals (0.9%) developed AV block; among them, 40 (69.0%) had no preceding MI or MACE. Two individuals (3.4%) of those with AV block died within 1 month of the diagnosis, and a total of 6 (10.3%) died within 1 year of the diagnosis. The characteristics associated with AV block are shown in Table 2. Older age (HR per 5-year increment, 1.47; 95% CI, 1.28-1.69; *P* < .001), hypertension (HR, 2.38; 95% CI, 1.36-4.16; *P* = .002), angina pectoris (HR, 5.45; 95% CI, 2.15-13.81; *P* < .001), MI (HR, 8.80; 95% CI, 3.47-22.30; *P* < .001), CHF (HR, 12.18; 95% CI, 4.30-34.47; *P* < .001), and higher levels of cholesterol (HR, 1.17; 95% CI, 1.06-1.27; *P* < .001), triglycerides (HR, 1.06; 95% CI, 1.04-1.10; *P* < .001), and fasting glucose (HR, 1.22; 95% CI, 1.13-1.32; *P* < .001) were each associated with a greater incidence of AV block. After multivariable adjustment, older age (HR per 5-year increment, 1.34; 95% CI, 1.16-1.54; *P* < .001), male sex (HR, 2.04; 95% CI, 1.19-3.45; *P* = .01), a higher systolic blood pressure (HR, 1.22; 95% CI, 1.10-1.34; *P* = .005), history of MI (HR, 3.54; 95% CI, 1.33-9.42; *P* = .01), history of CHF (HR, 3.33; 95% CI, 1.10-10.09; *P* = .03), and a higher fasting glucose level (HR, 1.22; 95% CI, 1.08-1.35; *P* = .001) were each independently associated with incident AV block (Table 2). In a sensitivity analysis including age, sex, and all other covariates exhibiting *P* < .10 in unadjusted analyses, systolic blood pressure (HR per 10–mm Hg increase, 1.26; 95% CI, 1.06-1.49; *P* = .007) and elevated glucose level (HR per 20-mg/dL increase, 1.22; 95% CI, 1.04-1.43; *P* = .01) maintained a statistically significant association with a heightened risk of AV block.

**Table 2.** Unadjusted and Multivariable-Adjusted Risk Factors Associated With Atrioventricular Block

Characteristic	Unadjusted Models		Multivariable Model^a^	
	HR (95% CI)	*P* Value	HR (95% CI)	*P* Value
Age^b^	1.47 (1.28-1.69)	<.001	1.34 (1.16-1.54)	<.001
Male sex	1.44 (0.86-2.24)	.16	2.04 (1.19-3.45)	.01
Height	1.0 (0.97-1.02)	.74	NA	NA
Weight	1.01 (0.99-1.03)	.17	NA	NA
Body mass index^c^	1.05 (0.99-1.12)	.10	NA	NA
Heart rate	1.00 (0.98-1.02)	.86	NA	NA
Blood pressure^d^				
Systolic	1.34 (1.22-1.48)	<.001	1.22 (1.10-1.34)	.005
Diastolic	1.40 (1.10-1.79)	.003	NA	NA
Hypertension	2.38 (1.36-4.16)	.002	NA	NA
Diabetes	4.44 (1.60-12.34)	.004	NA	NA
Angina pectoris	5.45 (2.15-13.81)	<.001	NA	NA
Myocardial infarction	8.80 (3.47-22.30)	<.001	3.54 (1.33-9.42)	.01
Congestive heart failure	12.18 (4.30-34.47)	<.001	3.33 (1.10-10.09)	.03
Blood pressure–lowering medication	1.95 (0.96-3.99)	.07	NA	NA
Smoking status^e^	1.26 (0.75-2.10)	.39	NA	NA
Alcohol consumption	1.00 (1.00-1.01)	.21	NA	NA
Cholesterol level^f^	1.17 (1.06-1.27)	<.001	1.08 (0.98-1.20)	.09
High-density lipoprotein level^f^	0.77 (0.54-1.10)	.14	NA	NA
Triglyceride level^f^	1.06 (1.04-1.10)	<.001	NA	NA
Fasting glucose level^f^	1.22 (1.13-1.32)	<.001	1.22 (1.08-1.35)	.001

Abbreviations: NA, not applicable; HR, hazard ratio.

^a^ Selection of covariates for multivariable models is explained in the Statistical Analysis subsection of the Methods section. Unless otherwise indicated, hazard is interpreted as the presence (vs absence) of each categorical variable or an increase of 1 unit of each continuous variable.

^b^ Interpreted as a hazard for every 5-year increase.

^c^ Calculated as weight in kilograms divided by height in meters squared.

^d^ Interpreted as a hazard for every 10–mm Hg increase.

^e^ Dichotomized into current smokers and former smokers vs nonsmokers.

^f^ Interpreted as a hazard for every 20-mg/dL increase.

**Figure 1.**
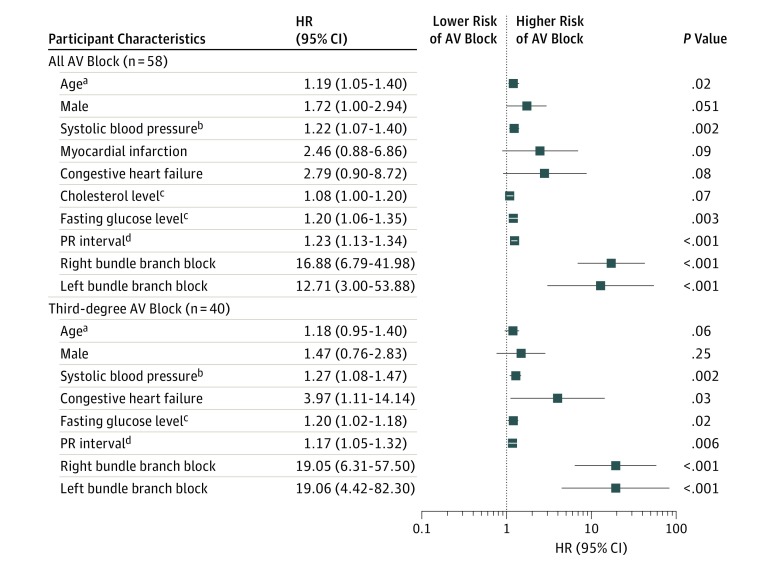
Multivariable Adjusted Hazard Ratios (HRs) for Incident Atrioventricular (AV) Block Incident AV block was defined as a hospitalization for second- or third-degree AV block during the follow-up. All covariates listed were included in multivariable models. Unless otherwise indicated, hazard ratios (HRs) were interpreted as a hazard for the presence (vs absence) of each categorical variable or for the increase of 1 unit of each continuous variable. To convert cholesterol to millimoles per liter, multiply by 0.0259; glucose level to millimoles per liter, multiply by 0.0555. ^a^Interpreted as a hazard for every 5-year increase. ^b^Interpreted as a hazard for every 10-mm Hg increase. ^c^Interpreted as a hazard for every 20-mg/dL increase. ^d^Interpreted as a hazard for every 10-millisecond increase.

**Figure 2.**
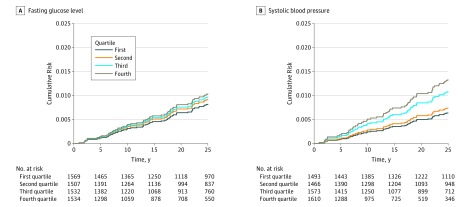
Cumulative Incidence of Atrioventricular Block Taking Death as a Competing Risk Into Account Data are shown according to quartiles of fasting glucose level and systolic blood pressure. The quartiles for systolic blood pressure were less than 126, 126 to 136, 137 to 152, and greater than 152 mm Hg. The quartiles for glucose level were less than 87.3, 87.3 to 93.1, 93.2 to 100.4, and greater than 100.4 mg/dL (to convert to millimoles per liter, multiply by 0.0555).

After adjusting for time-updated MACEs, neither a history of MI nor a history of CHF maintained a statistically significant association with incident AV block, and no other meaningful changes in associations with other covariates were observed. When ECG markers of less severe conduction disorders were examined, a longer PR interval (HR per 10-millisecond increase, 1.27; 95% CI, 1.18-1.34; *P* < .001), longer QRS duration (HR per 10-millisecond increase, 1.45; 95% CI, 1.21-1.74; *P* < .001), presence of right BBB (HR, 33.32; 95% CI, 14.17-78.37; *P* < .001), and presence of left BBB (HR, 23.16; 95% CI, 5.16-95.08; *P* < .001) were associated with incident AV block. When ECG-based evidence of conduction disease was added to the multivariable model, older age (HR per 5-year increment, 1.19; 95% CI, 1.05-1.40; *P* = .02), a higher systolic blood pressure (HR, 1.22; 95% CI, 1.07-1.40; *P* = .002), and an elevated fasting glucose level (HR, 1.20; 95% CI, 1.06-1.35; *P* = .003) maintained a statistically significant association with incident AV block, along with a longer PR interval (HR, 1.23; 95% CI, 1.13-1.34; *P* < .001), presence of right BBB (HR, 16.88; 95% CI, 6.79-41.98; *P* < .001), and presence of left BBB (HR, 12.71; 95% CI, 3.00-53.88; *P* < .001) (Figure 1). Restricting the outcome to third-degree AV block alone, higher systolic blood pressure (HR, 1.27; 95% CI, 1.08-1.47; *P* = .002), history of CHF (HR, 3.97; 95% CI, 1.11-14.14; *P* = .03), higher fasting glucose level (HR, 1.20; 95% CI, 1.02-1.18; *P* = .02), and the same ECG variables (HR, 1.17; 95% CI, 1.05-1.32; *P* = .006) were statistically significant risk factors (Figure 1). The unadjusted cumulative risk of AV block, according to quartiles of the 2 most directly modifiable risk factors, systolic blood pressure and fasting glucose level, is illustrated in Figure 2.

### Population-Attributable Risk

**Figure 3.**
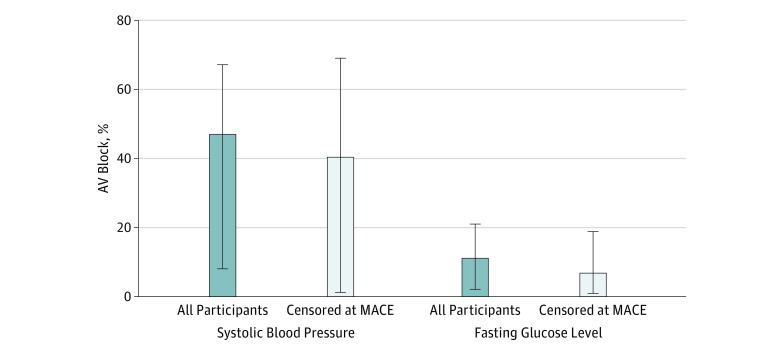
Multivariable-Adjusted Population-Attributable Risks of Systolic Blood Pressure and Fasting Glucose Level for Atrioventricular (AV) Block The dark blue bars represent the population-attributable risk for the listed covariates. The light blue bars represent the population-attributable risk after censoring participants from the model at the occurrence of major adverse coronary events (MACEs) (unstable angina pectoris, myocardial infarction, angioplasty, and/or coronary artery bypass graft). All models were adjusted for age, sex, history of myocardial infarction, history of congestive heart failure, and total cholesterol level. In addition, models for systolic blood pressure were adjusted for fasting glucose level, and models for fasting glucose level were adjusted for systolic blood pressure. Error bars represent 95% CIs.

In estimation of population-attributable risk, elevated systolic blood pressure may have been responsible for an estimated 47% (95% CI, 8%-67%) of all AV blocks, whereas elevated glucose level may explain an estimated 11% (95% CI, 2%-21%) of all AV blocks (Figure 3). After excluding time-updated coronary events, elevated systolic blood pressure continued to be statistically associated with 40% (95% CI, 1%-69%) of AV blocks, and elevated glucose level was associated with approximately 7% (95% CI, 1%-19%) of the outcomes observed.

## Discussion

An elevated systolic blood pressure and an elevated fasting glucose level were each associated with the development of AV block, even after adjusting for baseline and time-updated cardiovascular disease as well as ECG evidence of conduction disease. These results suggest that elevated blood pressure and blood glucose level may be associated with more than half of all cases of AV block.

Our study is, to our knowledge, the first community-based study to evaluate the association between common modifiable cardiovascular risks and the incidence of AV block. Therefore, selection bias that is commonly present in studies relying only on clinical information and experience with patients presenting with cardiovascular disease is not relevant. Considering the severity of the disease, we observed reasonably low 1-month and 1-year mortality rates after a diagnosis of AV block. Although ECG-based markers of conduction disease have been associated with future pacemaker implantation,^3,4,5,24^ associations between modifiable cardiovascular risks and second- and third-degree AV block (and the associated pacemaker implantations) have not previously been assessed.

Not surprisingly, MI was associated with the incidence of AV block. Temporary AV block associated with vagal effects and permanent AV block associated with structural damage of the conduction system are both known to occur as a result of MI.^2,25,26^ We similarly found that structural heart disease in the form of a history of MI or CHF was independently associated with AV block, but most AV blocks (69.0%) occurred without a preceding history of MI or MACE.

### Blood Pressure

The fact that a higher systolic blood pressure was associated with AV block even after adjusting for baseline MI and time-updated MACE suggests a possible causal effect that may not be mediated by infarction of cardiac tissue alone. Postmortem studies of individuals with AV block and no other prevalent cardiovascular disease demonstrate fibrosis of the conduction system.^2^ This fibrosis, also called *Lev disease*, is known to be age-related but is otherwise considered idiopathic.^27,28^ Hypertension and associated left ventricular hypertrophy are known to coexist with myocardial fibrosis.^29^ It is intriguing to speculate whether elevated blood pressure might also lead to local fibrosis infiltrating the AV conduction system.

Regardless of the mechanism, if the association between elevated systolic blood pressure and AV block was causal, our population-attributable risk calculations would suggest that almost half of all AV blocks could be connected to an elevated systolic blood pressure. Studies examining lowering of blood pressure to prevent conduction disease and AV block may be worthwhile, and perhaps at the very least this new information might help to encourage individuals with hypertension to receive and continue to use prescribed treatments.

### Fasting Glucose Level

Fasting glucose level was associated with AV block in all models, and 11% of the AV blocks could be attributed to it after adjusting for cardiovascular diseases and risks. Hyperglycemia, insulin resistance, and type 2 diabetes are known predispositions to coronary disease and MI, offering a possible explanation for the observed association.^30,31^ However, as with elevated systolic blood pressure, the association between elevated glucose level and incident AV block persisted even after adjusting for coronary events, suggesting that the true total effects may be substantially stronger than what was observed. Diabetes also causes multiple changes in the metabolism of cardiomyocytes, including increased lipotoxic effects due to aberrant use of fatty acids and increased reactive oxygen species production, both of which may lead to cardiomyocyte injury and cell death with resultant inflammation and fibrosis.^32,33^ Such processes may all predispose to conduction disease.

Of note, a diagnosis of hypertension and a diagnosis of diabetes were each statistically significantly associated with incident AV block; however, these associations were not retained in the multivariable models. This finding was almost certainly due to collinearity with the raw measurements of systolic blood pressure and blood glucose levels, which likely overwhelmed the simpler, dichotomous risk factors because of their continuous nature (providing more power and precision to detect a difference). However, given that these diagnoses and measurements in many ways illustrate the same general pathophysiology, it would be inappropriate to conclude, for example, that a diagnosis of hypertension or a diagnosis of diabetes was not an important risk factor for AV block.

### Strengths and Limitations

To our knowledge, this study is the largest to date to examine risk factors for incident AV block and the only community-based study to be used for this purpose. Our study population was not limited to individuals seeking medical care, as is typical in medical record review studies or studies using administrative databases. Particularly comprehensive baseline covariate ascertainment was performed in a uniform fashion according to a prespecified protocol. This study also leverages the hospital admission data registry in Finland, which captures all hospitalizations. Because clinically relevant AV block would very likely result in hospitalization, we expect that outcome ascertainment was particularly sensitive. Finally, follow-up extending to 32 years enabled us to collect a sufficiently large number of outcomes to detect several statistically significant associations and to adjust for important time-updated events.

One limitation of this study is that it was performed in a solely white population, and the extrapolation of its results to other populations should be performed with caution. In addition, the observational nature of our study does not allow us to draw any conclusions about the causal relationship between the cardiovascular risks observed and incidence of AV block. Atrioventricular block ascertainment for the study began in 1987, leaving a 7- to 9-year gap during which only data regarding death and MACEs were available. Although this restriction might primarily reduce our power to detect significant associations, risk factors for particularly more severe forms of conduction disease might have been missed. The *ICD* codes do not differentiate between Mobitz type I and II blocks, and the pathophysiology and clinical relevance of these 2 forms of AV block can be very different.^34,35^ However, our analysis of only those with third-degree AV block did not reveal any meaningful differences. Temporary AV block is known to occur at the time of myocardial ischemia or MI,^2^ and we cannot exclude the possibility that some of the outcomes observed were owing to that phenomenon. However, analyses adjusting for time-updated MACEs and population-attributable risk calculations after censoring all patients with a MACE continued to demonstrate that an elevated systolic blood pressure and blood glucose level were independently associated with AV block.

We acknowledge possible disagreement regarding the optimal approach to conducting multivariable analyses to determine the most important risk factors for a given outcome. For example, according to a recent set of guidelines, our select stepwise approach may not always be appropriate but is considered suitable in the absence of previous studies to identify the likely covariates for inclusion.^36^ Given the relative dearth of research in this area, we believe the approach used was most appropriate; furthermore, in a sensitivity analysis using broader inclusion of covariates, the associations for elevated systolic blood pressure and elevated glucose level remained statistically significant. A next natural step might be to conduct studies specifically to construct and test a risk prediction score and to provide evidence that might reveal mechanisms contributing to the disease process and prevention strategies. Although such a step is beyond the scope of the current study, our finding that readily accessible data may be identified as important to future AV block risk might motivate even larger cohorts, consortia of cohorts, or planned prospective studies to institute this next step.

## Conclusions

Atrioventricular block is associated with multiple known cardiovascular risk factors and conditions. In this study, the common, easily measured, and modifiable risk factors of an elevated systolic blood pressure and a higher fasting glucose level were independently associated with AV block. Taken together, these 2 directly modifiable variables potentially explain more than half of all AV blocks in a community-based population. Effective treatment of hypertension and maintenance of normal blood glucose levels may be useful strategies in preventing AV block.

## Notes

Supplement. **eTable.** Characteristics of the Study Population and Individuals Excluded Due to Early Death (n = 854), Missing/Poor Quality ECG (n = 200), or Other Missing Covariate (n = 17)
