# Supplementary material for: Risk Factors Associated With Atrioventricular Block
Source: JAMA Netw Open. 2019 May 24;2(5):e194176. doi: 10.1001/jamanetworkopen.2019.4176 (PMC6632153; doi:10.1001/jamanetworkopen.2019.4176)
Supplement: Supplement. — eTable. Characteristics of the Study Population and Individuals Excluded Due to Early Death (n = 854), Missing/Poor Quality ECG (n = 200), or Other Missing Covariate (n = 17) [file jamanetwopen-2-e194176-s001.pdf]

Supplementary Online Content

Kerola T, Eranti A, Aro AL, et al. Risk factors associated with atrioventricular block. *JAMA Netw Open*. 2019;2(5):e194176. doi:10.1001/jamanetworkopen.2019.4176

eTable. Characteristics of the Study Population and Individuals Excluded Due to Early Death (n = 854), Missing/Poor Quality ECG (n = 200), or Other Missing Covariate (n = 17)

This supplementary material has been provided by the authors to give readers additional information about their work.

©2019 Kerola T et al. *JAMA Network Open*

eTable. Characteristics of the Study Population and Individuals Excluded Due to Early Death (n = 854), Missing/Poor Quality ECG (n = 200), or Other Missing Covariate (n = 17)

**Characteristics**	**Study participants (n=6146)**	**Individuals excluded (n=1071)**	**p**
Age, mean (SD), years	49.2 (12.9)	63.8 (14.5)	**<.001**
Sex, n (%), male	2697 (44)	625 (59)	**<.001**
Height, mean, (SD), cm	165.8 (9.4)	164.7 (9.9)	**<.001**
Weight, mean, (SD), kg	71.3 (13.0)	70.2 (13.6)	**<.001**
Body mass index, mean (SD), kg/m²	25.9 (4.1)	25.8 (4.2)	.054
Heart rate, mean (SD), beats per minute	67.5 (13.2)	73.5 (16.4)	**<.001**
Systolic blood pressure, mean (SD), mmHg	141.4 (21.8)	154.8 (26.7)	**<.001**
Diastolic blood pressure, mean (SD), mmHg	86.6 (11.3)	88.4 (12.8)	**<.001**
Hypertension, n (%)	3510 (57)	820 (77)	**<.001**
Diabetes, n (%)	225 (4)	187 (18)	**<.001**
Angina pectoris, n (%)	244 (4)	152 (14)	**<.001**
Myocardial infarction, n (%)	160 (3)	130 (12)	**<.001**
Congestive heart failure, n (%)	123 (2)	136 (13)	**<.001**
Blood pressure lowering medication, n (%)	791 (13)	252 (24)	**<.001**
Alcohol consumption, median (IQR), grams/week	7.0 (0–49)	0 (0–24)	**<.001**
Smoking status			**<.001**
non smoker, n (%)	3473 (57)	526 (49)	
ex-smoker, n (%)	1264 (21)	241 (23)	
current smoker, n (%)	1409 (23)	294 (27)	
Cholesterol, mean (SD), mg/dL	267 (54)	271 (58)	.051
High-density lipoprotein, mean (SD), mg/dL	66 (15)	62 (15)	**<.001**
Triglycerides, mean (SD), mg/dL	58 (39)	73 (22)	**<.001**
Fasting glucose, mean (SD), mg/dL	95 (22)	108 (47)	**.001**

Data are presented as means ± SD, medians (interquartile range [IQR]) or numbers (percentage).P values are based on Student's t-test, chi-square test and Mann-Whitney U test. To convert cholesterol, high-density lipoprotein and triglycerides from mg/dL to mmol/l multiply with 0.02586. To convert fasting glucose from mg/dL to mmol/l multiply with 0.05556.

©2019 Kerola T et al. *JAMA Network Open*
